# High-Sulfated Glycosaminoglycans Prevent Coronavirus Replication

**DOI:** 10.3390/v14020413

**Published:** 2022-02-17

**Authors:** Stephanie Möller, Janine Theiß, Thaira I. L. Deinert, Karoline Golat, Julian Heinze, Daniela Niemeyer, Ralf Wyrwa, Matthias Schnabelrauch, Elke Bogner

**Affiliations:** 1 INNOVENT e.V., Biomaterial Department, 07745 Jena, Germany; sm@innovent-jena.de (S.M.); ralf.wyrwa@ikts.fraunhofer.de (R.W.); ms@innovent-jena.de (M.S.); 2 Institute of Virology, Charité-Universitätsmedizin Berlin, 10117 Berlin, Germany; theiss.janine@web.de (J.T.); thaira.deinert@charite.de (T.I.L.D.); karoline.golat@charite.de (K.G.); julian.heinze@charite.de (J.H.); daniela.niemeyer@charite.de (D.N.); 3 German Center for Infection Research (DZIF), 10117 Berlin, Germany

**Keywords:** bovine coronavirus, SARS-CoV-2, high-sulfated glycosaminoglycans, antiviral activity

## Abstract

Coronaviruses (CoVs) are common among humans and many animals, causing respiratory or gastrointestinal diseases. Currently, only a few antiviral drugs against CoVs are available. Especially for SARS-CoV-2, new compounds for treatment of COVID-19 are urgently needed. In this study, we characterize the antiviral effects of two high-sulfated glycosaminoglycan (GAG) derivatives against SARS-CoV-2 and bovine coronaviruses (BCoV), which are both members of the Betacoronavirus genus. The investigated compounds are based on hyaluronan (HA) and chondroitin sulfate (CS) and exhibit a strong inhibitory effect against both CoVs. Yield assays were performed using BCoV-infected PT cells in the presence and absence of the compounds. While the high-sulfated HA (sHA3) led to an inhibition of viral growth early after infection, high-sulfated CS (sCS3) had a slightly smaller effect. Time of addition assays, where sHA3 and sCS3 were added to PT cells before, during or after infection, demonstrated an inhibitory effect during all phases of infection, whereas sHA3 showed a stronger effect even after virus absorbance. Furthermore, attachment analyses with prechilled PT cells revealed that virus attachment is not blocked. In addition, sHA3 and sCS3 inactivated BCoV by stable binding. Analysis by quantitative real-time RT PCR underlines the high potency of the inhibitors against BCoV, as well as B.1-lineage, Alpha and Beta SARS-CoV-2 viruses. Taken together, these results demonstrated that the two high-sulfated GAG derivatives exhibit low cytotoxicity and represent promising candidates for an anti-CoV therapy.

## 1. Introduction

Coronaviruses (CoVs), a member of the large family *Coronaviridae*, are RNA viruses with the largest coding capacity identified among RNA viruses. CoVs infect amphibians, birds and mammals including humans. The first description goes back to the 1960s where J. Almeida has performed the first electron microscopy analysis [1]. One subfamily of *Coronaviridae* is *Orthocoronavirinae*, which consists of the four genera: Alpha-, Beta-, Gamma-, and Deltacoronaviruses (International Committee on Taxonomy of Viruses). While Alpha- and Betacoronaviruses only infect mammals, Gamma- and Deltacoronaviruses infect birds and only a few can also infect mammals [2]. In this study, we used the two representative members of the Betacoronavirus genus, the severe acute respiratory syndrome (SARS)-CoV-2 and the bovine coronavirus (BCoV), as models for compound testing.

BCoV is a member of the Betacoronavirus lineage A (subgenus Embecovirus) and belongs, together with the human CoV (HCoV) OC43, to the species *Betacoronavirus 1*. BCoV causes pneumoenteric diseases and leads to severe losses in cattle farming industry [3,4,5].

SARS-CoV-2 is a human pathogenic CoV and a lineage B (subgenus Sarbecovirus) member of the Betacoronavirus genus, which caused a pandemic in 2020 with tremendous impact on public health, society and economy. Together with SARS-CoV-2, HCoVs 229E, OC43, NL63 and HKU1 are associated with upper respiratory tract infections and cause a mild infection—known as the common cold. Under some circumstances, HCoVs can spread to lungs or other organs [6]. Furthermore, in the last two decades it has been demonstrated that CoVs can cause life-threatening diseases. The emergence of SARS-CoV in 2002, SARS-CoV-2 in 2019 and the Middle East respiratory syndrome (MERS) CoV in 2012 showed that coronaviruses can be associated with serious respiratory diseases [7,8,9,10]. Even after a decade of research, no specific treatment against the highly pathogenic CoVs is available.

The CoV genome encodes two-thirds non-structural and one-third structural proteins. The structural proteins consist of the membrane M, the envelope E, the spike S and the nucleocapsid N proteins, and are involved in virus entry and maturation [11,12,13]. As with all representatives of the Embecovirus subgenus, BCoVs encode an additional structural protein, the hemagglutinin esterase HE [14].

The initial contact of viruses with the host cell is mediated through binding of viral envelope proteins to glycans, heparan sulfate proteoglycans (HSPG). HSPGs are localized on the cell surface and the extracellular matrix of all mammalian cells [15]. This attachment facilitates binding to the cellular receptor resulting in viral entry. Recently, Clausen et al. [16] demonstrated that SARS-CoV-2 spike protein interacts with HSPG. By binding to HSPG, the receptor-binding domain (RBD) changes its structure into an open conformation, which is able to bind to the angiotensin converting enzyme (ACE-2) receptor. BCoV uses 9-O-acetylated sialic acids and heparan sulfate for virus attachment [17]. In addition, HLA class I molecules were shown to serve as receptors for BCoV entry [18]. These findings indicate that HSPGs are important co-factors for coronaviruses and potential targets for antiviral therapy.

Several other naturally and chemically sulfated polysaccharides and glycosaminoglycans (GAGs) have already been described for their potent inhibitory effects on virus infections [19,20]. It has been reported that various carrageenans, a family of natural linear, sulfated polysaccharides extracted from red seaweeds, inhibit the infection of human viral pathogens as papilloma [21] and influenza A [22] viruses. Sulfated derivatives of *E. coli* K5 capsular polysaccharides are potent inhibitors of human Cytomegalovirus [23]. The natural GAG chondroitin-4,6-sulfate (formerly known as chondroitin sulfate E) has been considered as an antiviral agent against T-cell leukemia virus type 1 [24] and the flavivirus dengue by interaction with the envelope (E) protein of the latter virus [25]. In a previous publication an antiviral activity of sulfated hyaluronan against Herpes simplex virus type 1 could be shown [26].

In this study, we designed and synthesized high-sulfated GAGs, based on the naturally non-sulfated hyaluronan (HA) and the low-sulfated chondroitin sulfate (CS) that might be able to block the interaction with HSPG. We characterized their antiviral activity against BCoV and SARS-CoV-2 as models for Betacoronaviruses.

## 2. Materials and Methods

### 2.1. Cells and Virus

Sheep epithelial proximal tubular (PT) cells CCLV-RIE 0011 (Collection of Cell Lines in Veterinary Medicine (CCLV), Friedrich-Loeffler-Institut, Greifswald, Germany) and human colon adenocarcinoma cell line Caco-2 (ECACC) were grown in Dulbecco’s minimal essential medium (DMEM) supplemented with 10% fetal bovine serum (FBS), 2 mM glutamine and gentamicin (60 µg/mL). Sequencing of the whole BCoV genome demonstrated 99.17% identity with strain “Mebus” (U00735.2) and 99.26% with strain “Kakegawa (AB35470.1). Both belong to group 1 [27]. Infection with bovine coronavirus (BCoV) was carried out as described before [28]. The stocks of BCoV were titrated by plaque reduction assays in PT cells. SARS-CoV-2 infection experiments were done with the SARS-CoV-2 strains BetaCoV-19/Munich/ChVir-984/2020 (GISAID: EPI_ISL_406862, B.1 lineage), hCoV-19/Germany/BW-ChVir21528/2020 (GISAID: EPI_ISL_754174, B.1.1.7 lineage) and hCoV-19/Germany/BW-ChVir22131/2021 (GISAID: EPI_ISL_862149, B.1.351 lineage). Virus stock production was conducted on Vero E6 cells for 3 days. Supernatant was purified by ultrafiltration (Vivaspin 20, molecular weight exclusion <100 kDa), diluted in virus preservation medium (OptiPro containing 0.5% gelatine) and aliquots were stored at −80 °C. All stocks were plaque titrated and fully sequenced prior to use.

### 2.2. Polymers and Chemicals for Synthesis

Hyaluronan (HA, from Streptococcus sp., Mw (weight-average molecular weight) 9.3 × 10^5^ g mol^−1^) as starting material for high-sulfated HA (sHA3) and chondroitin sulfate (CS, from bovine tracheal, a mixture of 70% chondroitin-4-sulfate and 30% chondroitin-6-sulfate, Mw 2.16 × 10^4^ g mol^−1^, D.S. (degree of sulfation) = 0.9) as educt for high-sulfated CS (sCS3) was purchased from Kraeber (Ellerbek, Germany). Sulfur trioxide/dimethylformamide complex (SO_3_-DMF, 47% active SO_3_) and sulfur trioxide/pyridine complex (SO_3_-pyridine, active SO_3_ ~ 48–50% pract., ≥45% SO_3_) were obtained from Fisher Scientific (Schwerte, Germany).

### 2.3. Synthesis of High-Sulfated GAGs

The synthesis of sulfated GAGs was previously described. Briefly, the syntheses were performed as follows: for the synthesis of sHA3, as starting material, a thermally degraded HA (Mw 4.63 × 10^4^ g mol^−1^) was used. The thermal degradation of HA was performed in an autoclave at 130 °C for 200 min according to a reported protocol [29]. The degraded HA was sulfated with SO_3_-pyridine [30,31,32] using a ratio of polymeric OH-group/SO_3_ of 1: 15. The reaction time was one hour at room temperature. Prior to sulfation, CS was purified by dialysis and then sulfated with SO_3_-DMF (OH/SO_3_ = 1: 20) [33].

The polymers were purified by dialysis, first against aqueous 0.025 molar NaHCO_3_-solution and subsequently against de-ionized water. In the next step, the samples were subjected to freeze-drying and drying at a vacuum pump.

### 2.4. Characterisation of High-Sulfated GAGs

The respective degree of sulfation (D.S., the average numbers of sulfate groups per disccharide repeating unit) of the high-sulfated GAGs was determined by elemental analysis, and the molecular weight and the dispersity was estimated by gel permeation chromatography (GPC) equipped with laser light scattering-, refraction index- and UV-detection. The sulfate group distributions within the repeating disaccharide unit of the GAG were detected by high-resolution ^13^C nuclear magnetic resonance (NMR) [26,34].

**Figure 1:**
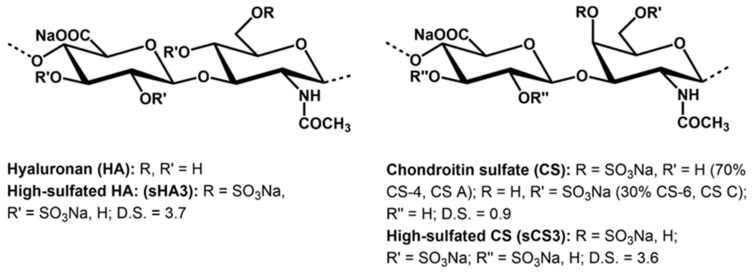
Structure of native and chemically sulfated GAGs (the used native chondroitin sulfate is a mixture of chondroiti-4-sulfate (CS A) and chondroitin-6-sulfate (CS C)). The D.S. (degree of sulfation) values are the average numbers of sulfate groups per disaccharide repeating unit.

Structural data of the prepared GAG derivatives (sHA3, sCS3) are shown in Figure 1.

The compounds were dissolved in Aqua bidest. Stock solutions of 20 mg/mL and stored at 4 °C prior to use.

### 2.5. Plaque Reduction Assay

PT cells (6.5 × 10^4^) were seeded in 24-well plates and infected with BCoV (MOI 0.00005). After 1 h post-infection (hpi) the inoculum was replaced by 2 mL of 2.4% (*w*/*v*) Avicel RC-581 (FMC BioPolymer, Belgium) containing 2 × MEM, 5% FBS and various concentrations of sHA3 (0.0, 0.1, 0.3, 0.4, 0.6, 0.9 µM) or sCS3 (0.0, 0.4, 0.9, 1.0, 2.0, 3.0 µM). After incubation for four days at 37 °C, the cells were fixed with 6% formalin in PBS pH 7.4 prior to staining with 0.2% (*w*/*v*) crystal violet in 20% EtOH for 15 min at room temperature. Plaques were counted by using a light microscope (Axiovert 10, Carl Zeiss Microscopy, Jena, Germany) and compound effects were calculated by comparing compound-treated cells versus untreated cells.

### 2.6. Cytotoxicity Determination

PT or Caco-2 cells were seeded in 96-well plates (5 × 10^4^) and grown until 70% confluence. Subconfluent PT cells were incubated with several concentrations of sHA3 or sCS3 (50, 150, 250, 350, 450, 550, 650, 700 µM) in a final volume of 100 µL for four days at 37 °C. Cytotoxicity (50% cytotoxic concentration, CC_50_) profiling of the compounds was determined by the use of Cell Proliferation Kit II (XTT, Roche) as recommended by the manufacturer. Briefly, after the incubation time, 50 µL of the labelling solution (XTT labeling reagent and electron coupling reagent, 50: 1) were added to the cells, followed by incubation of 4 h at 37 °C. The absorbance of 492 nm with a reference wavelength of 650 nm was measured using an ELISA reader.

### 2.7. Yield Assay

Confluent PT cells (5 × 10^4^ cells per well) were seeded in 24-well plates. Confluent PT cells were treated for 30 min with 2.5 µM and 5 µM sHA3 or sCS3 or left untreated. Three washing steps with PBS before infection with BCoV (MOI 0.00005) removed compounds. At 24, 48, 72, and 96 hpi, supernatants were harvested and frozen at −80 °C. After collection of all time points, supernatants were thawed and transferred to PT cells grown on 12-well plates, and after four days titers were determined by plaque reduction assay. Fixation and staining were described by plaque reduction assay.

### 2.8. Effects of Pre-, Co- and Post-Treatment

The treatment analysis was performed as described before [35] (time of addition analysis). Briefly, PT cells were seeded in 24-well plates (5 × 10^4^ cells per well) and grown until confluence. sHA3 (0.2996 µM) or sCS3 (1.7156 µM) were added: (i) 30 min before infection (before inf.) and the compound was removed by three washing steps with PBS prior to infection, (ii) during infection (during inf.) or (iii) after infection (after inf.) in the avicel overlay. Untreated cells served as the control. Infection was performed with BCoV (MOI 0.00005) for 90 min at 37 °C. Then, inoculum was removed by three washing steps with PBS. Afterwards, 2 mL of 4% (*w*/*v*) Avicel RC-581 was added and the plaque assay was performed as described above.

### 2.9. Attachment Assay

PT cells were seeded in 12-well plates (2 × 10^5^ cells per well). Prechilled confluent cells were treated with 0.2996 µM of sHA3, 1.7156 µM of sCS3 or left untreated for 30 min at 4 °C. The compounds were removed, and the cells were washed three times with prechilled PBS prior to infection with prechilled BCoV (MOI 0.00005) for 2 h at 4 °C. Thereafter, unattached virus was rinsed from the cells and the cells were subjected to plaque reduction assays at 37 °C for four days [35].

### 2.10. Penetration Assay

PT cells were seeded in 12-well plates (2 × 10^5^ cells per well). Confluent cells were cooled down to 4 °C and infected with BCoV (MOI 0.00005) for 2 h at 4 °C. After inoculums were removed by three washing steps with pre-cooled PBS, cells were treated with 2.5, 5 or 10 µM sHA3 or sCS3, 10 µg/mL heparin or medium for 10 min at 37 °C. The compound dilutions were aspirated and the cells were rinsed with Tris/HCl pH 3.0 in order to inactivate not penetrated virus. The cells were subjected to plaque reduction assays and analyzed after four days.

### 2.11. Effect of GAGs on Virus Inactivation

BCoV (MOI 0.005) was either incubated with 10 µM sHA3 or sCS3 or with medium for one hour at 37 °C. Untreated virus and virus–compound samples were diluted 1: 1000 with culture medium and the cells were infected for 1 h at 37 °C. After washing the cells three times with PBS, the cells were subjected to plaque reduction assays for three days.

### 2.12. SARS-CoV-2 Infection

Caco-2 cells (300,000 cells per well) were seeded to 24-well plates and infected with SARS-CoV-2 isolate one day after seeding. Culture medium was removed, cells were washed once with PBS and cells were inoculated with virus at the desired concentration (MOI 0.001, 0.0001). Adsorption was done at 37 °C for 1 h in a 5% CO_2_ atmosphere. After adsorption, the virus dilution was removed and the cells were washed twice with PBS to remove unbound particles, and wells were refilled with virus infection medium (DMEM supplemented with 2% FCS, 1% of 100× non-essential amino acids solution and 1% of 100 mM sodium pyruvate; [36]). Samples were taken from the supernatant at the indicated time points as indicated in the section Real-time RT PCR assays. Compounds (sHA3, sCS3 or diluent control) were diluted in different concentrations (two-fold dilution series ranging from 10 to 0.6 µM) in virus infection medium and added to the cells prior to and during infection. All SARS-CoV-2-related work was performed under biosafety level 3 conditions with enhanced respiratory personal protection equipment.

### 2.13. BCoV Infection

PT cells were seeded in 24-well plates (1.62 × 10^5^ cells per well). Confluent cells were treated with 1.25, 2.5, 5.0, 10 and 20 µM of sHA3 and sCS3, resp., for 1 h at 37 °C before infection. The inoculum was removed, stored at 4 °C and the cells were washed with PBS prior to infection with BCoV (MOI 0.00001, 0.00005 and 0.0005). After 1 h, the virus suspension was aspirated, cells were washed with PBS and 500 µL DMEM with 2% FBS, and 150 µL of the inhibitor solutions was added. Samples were taken at 24 and 48 hpi from the supernatant as indicated in the section Real-time RT PCR assays.

### 2.14. Real-Time RT PCR Assays

Quantitative real-time RT PCR assays were conducted to determine the concentration of viral RNA after compound treatment. At 24 and 48 hpi, a 50 µL sample was taken from the supernatant of infected and compound-treated PT and Caco-2 cells and diluted in 300 µL of MagNA Pure 96 External Lysis Buffer (Roche). Supernatant from SARS-CoV-2 infected cells was additionally heat-inactivated for 10 min at 70 °C before viral RNA was extracted using the MagNAPure 96 Small Volume Kit (Roche). RNA was subjected to either Invitrogen Superscript III one-step real-time RT PCR using E-gen assay for detection of SARS-CoV-2 [37] or Qiagen OneStep RT-PCR Kit and a nucleocapsid gene-specific assay for detection of BCoV RNA [38]. These quantitative PCRs amplified the nucleocapsid gene (N) of BCoV and the envelope gene (E) of SARS-CoV-2. The specific primers and probes are indicated in Table 1.

Quantification of BCoV viral RNA was performed by nucleocapsid gene- or envelope gene-specific assay. Briefly, 5 µL of extracted RNA sample was added to a master mix containing 5 µL 5× OneStep Qiagen buffer, 10 mM dNTPs, 10 µM sense primer, 10 µM antisense primer, 10 µM probe and 1 µL Qiagen enzyme mix in a final volume of 25 µL. The PCR started at 50 °C for 30 min, followed by activation of the polymerase at 95 °C for 15 min. A total of 45 cycles of amplification (95 °C for 15 s, 60 °C for 30 s) and 40 °C for 30 s were performed in a Light Cycler 480 (Roche Diagnostics, Switzerland). The probes were normalized against an internal RNA positive control. E gene assay for quantification of SARS-CoV-2 viral RNA was performed as previously described in Corman et al., 2020 [37].

### 2.15. Statistical Analysis

All experiments were performed in duplicate or triplicate in three biological replicates. Data were expressed as ± standard deviation (SD). Differences in the drug addition experiments were evaluated by Student’s *t*-test; *p*-values are denoted as follows: * *p* < 0.05, ** *p* < 0.01, *** *p* < 0.001. Variances of the means were evaluated by analysis of variance (ANOVA) followed by the Dunnett’s multiple comparison test using the software GraphPad Prism V 8.0 (GraphPad Software Inc., La Jolla, CA, USA). Prism determined multiplicity adjusted *p*-values by the entire family comparison [39]. *P*-values are denoted as follows: * *p* < 0.0332, ** *p* < 0.0021, *** p < 0.0002, **** *p* < 0.001. The antiviral determination was shown as the statistical value the coefficient of variation (CV). This is a standardized measure of a frequency distribution. CV is defined as the ratio of the standard deviation to the mean value.

## 3. Results

### 3.1. Synthesis of Sulfated GAG Derivatives

The homogeneously performed sulfation of hyaluronan (HA) and chondroitin sulfate (CS) using sulfur trioxide/amin complexes (SO_3_-DMF and SO_3_-pyridine, resp.) leads to products with a high number of sulfate groups per repeating disaccharide unit (D.S.). In the case of sHA3, a D.S. value of 3.7, and for sCS3 a D.S. of 3.6 was received. Having in mind that the unmodified GAG has four free OH-groups per anhydrodisaccharide units this means that most of the hydroxyl groups of the molecules are sulfated. ^13^C-NMR studies confirm that the primary hydroxyl groups at C-atom 6 of the N-acetylglycosamine unit are completely sulfated, with the residual free OH-groups distributed among the secondary OH groups present in the molecule.

The structural and analytical data of the prepared GAG derivatives (sHA3, sCS3) are summarized in Figure 1 and Table 2, respectively.

### 3.2. Antiviral Activity

**Figure 2:**
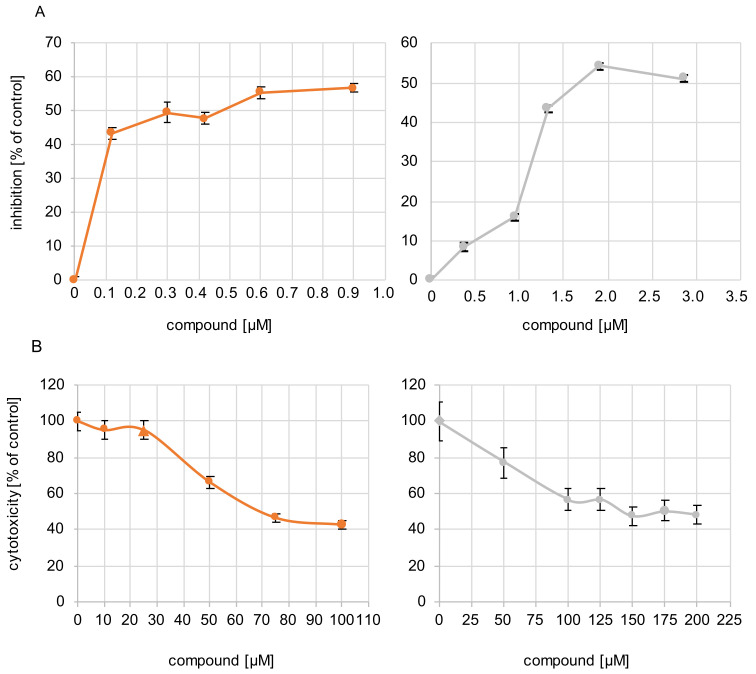
Antiviral activity of the compounds and their cytotoxicity. (**A**) Cells were infected with BCoV (MOI 0.00005) for 1 h. After infection, the inoculum was replaced by Avicel overlay containing increasing concentrations of sHA3 (orange) or sCS3 (grey). At 4 d p.i., plaque reduction assay was performed. Plaque reduction is indicated as inhibition as a percentage of PFU obtained in the absence of compounds. The mean 50% effective concentrations (EC_50_) range from 0.2996 µM for sHA3 and 1.7156 µM for sCS3. Results were obtained from three independent experiments. Error bars represents the coefficient of variation. (**B**) Various concentrations of sHA3 or sCS3 (µM) were added to PT cells. At 24 h, XTT cell proliferation assay was performed. Values are represented as the percentage of untreated control cells. Data are mean values from three independent experiments. Error bars represent standard deviation.

The antiviral efficacy of sHA3 and sCS3 against bovine coronavirus (BCoV) was determined by measurement of viral plaque formation (plaque reduction assay) and measurement of growth kinetics. The 50% effective concentrations (EC_50_) against BCoV obtained by plaque reduction ranged from 0.2996 µM for sHA3 to 1.7156 µM for sCS3 (Table 3; Figure 2). These results demonstrated that both compounds were active against BCoV in the viral plaque assay, and that sHA3 appeared to be the most active compound. In addition, the compounds were not cytotoxic at concentrations lower than 69 µM or 141 µM (Table 3; Figure 2), thus demonstrating that the effects were based on mainly the antiviral activity. The selectivity index (SI) is the ratio of the cytotoxicity of the compound against its effective concentration. In general, an SI ≥ 10 indicates a selective bioactive compound. The higher the SI ratio of a compound, the higher the efficacy in inhibition of virus replication is. Our results demonstrated that sHA3 (SI = 232.68) is more efficient compared to sCS3 (SI = 82.39), but both are bioactive.

**Figure 3:**
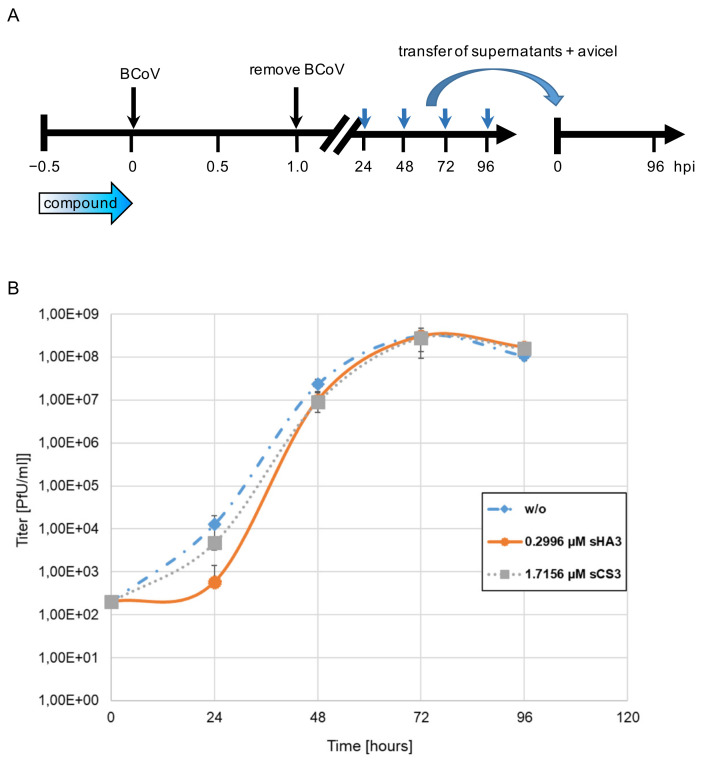
Growth curves in the presence of high-sulfated GAGs. (**A**) Diagram of the experimental settings. (**B**) PT cells were infected with BCoV (MOI 0.00005) in the absence (*w*/*o*) or presence of 0.2996 µM sHA3 or 1.7156 µM sCS3. At each time point, supernatants were harvested and titers determined on PT cells. Given are mean values and corresponding SD of three independent experiments.

To confirm the antiviral effects of the compounds on viral replication yield assays were performed with cell cultures treated for 30 min with 0.2996 sHA3 or 1.7156 µM sCS3 or left untreated, followed by infection with BCoV (MOI 0.00005) (Figure 3A). Viral titers in the supernatant were determined by plaque assay at the indicated time points. The virus yield was reduced about 1.3 log at 24 hpi for sHA3 and 0.5 log at 24 hpi for sCS3 (Figure 3B). The effect disappeared at later time points, indicating that the compounds block an early step in the infection cycle.

### 3.3. Effects of Pre-, Co- and Post-Treatment

**Figure 4:**
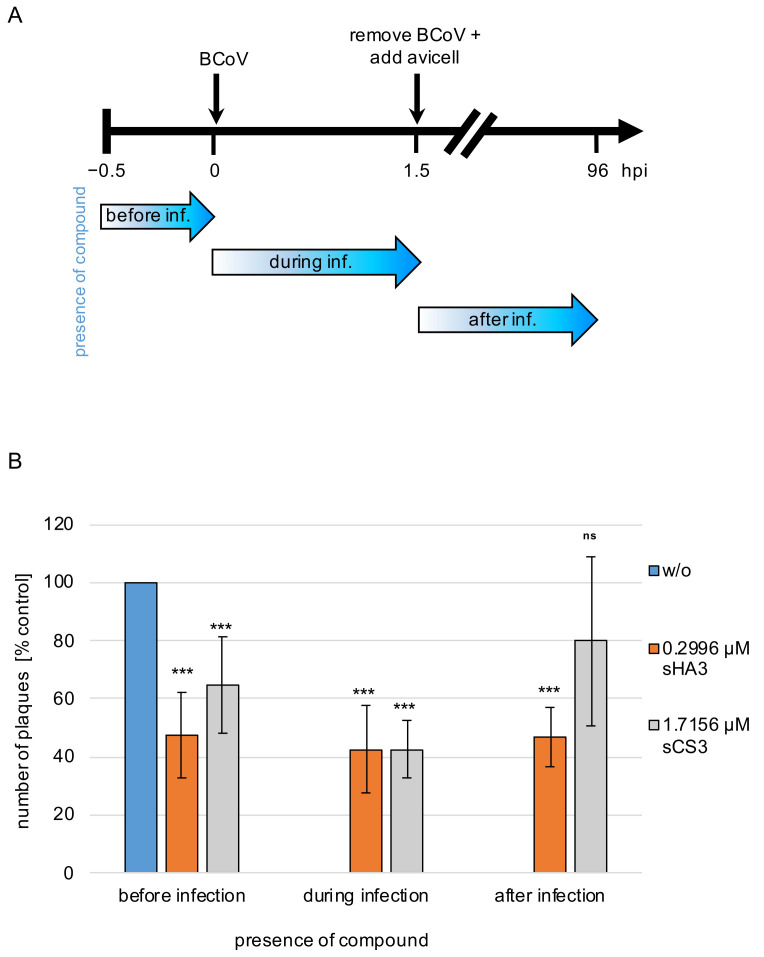
Effects of pre-, co- and post-treatment. (**A**) Diagram of the experimental settings. (**B**) PT cells treated with 0.2996 µM sHA3 or 1.7156 µM sCS3 before, during and after infection or left untreated (*w*/*o*) were subjected to plaque reduction assays. Shown are mean values and SD of three independent experiments. *** *p* < 0.001; ns, not significant.

To determine the phase of viral replication that is sensitive to the compounds, the effect of time-dependent drug addition was analyzed. PT cells were infected with BCoV (MOI 0.00005) and treated with the substances at EC_50_ (sHA3, 0.2996 µM; sCS3, 1.7156 µM) at different times of infection or left untreated. At day four post infection the effect on viral replication was quantified by plaque reduction (Figure 4A). Preincubation of the cells resulted in plaque reduction of approximately 57% for sHA3 and 38% for sCS3 compared to the untreated control (Figure 4B). Similar observations were seen if sHA3 was present during or after viral adsorption (Figure 4B). In contrast, sCS3 showed the most pronounced reduction if the compound was added during viral adsorption (58%) and the weakest (20%) by addition after viral adsorption (Figure 4B). These results revealed that sHA3 could be useful not only for prophylactic treatment but also as a therapy for SARS-CoV-infected patients.

### 3.4. Effect on Viral Attachment to the Host Cells

**Figure 5:**
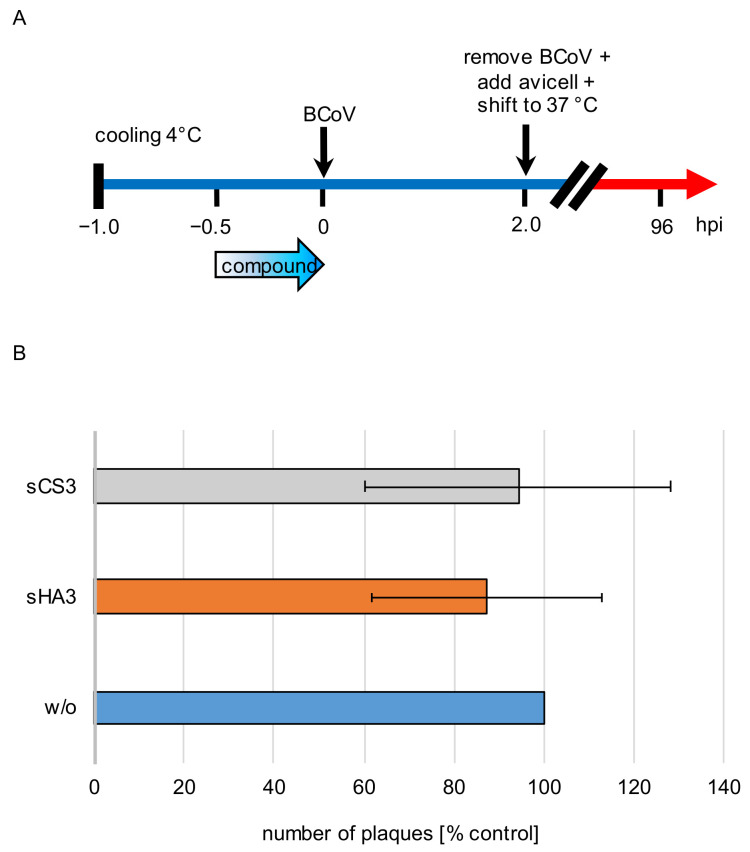
Effects of the high-sulfated GAGs on attachment. (**A**) Diagram of the experimental settings. (**B**) Prechilled PT cells were treated with 0.2996 µM sHA3 or 1.7156 µM sCS3 or left untreated (*w*/*o*) for 30 min at 4 °C. After removing the inhibitors, cells were infected with prechilled BCoV (MOI 0.00005) for 2 h at 4 °C. Three washing steps aspirated unattached virus, cells were overlaid with Avicel RC-581 and incubated for 4 days. Error bars on the histogram are SD from three independent experiments.

In order to analyze the effect of both compounds on viral attachment, prechilled PT cells were treated with 0.2996 µM sHA3, 1.7156 µM sCS3 or left untreated on ice for 30 min at 4 °C. After removal of the inhibitors, cells were infected with prechilled BCoV (MOI 0.00005) for 2 h at 4 °C. Unattached virus was removed by three washing steps. After four days of infection cells were subjected to plaque reduction assays (Figure 5A). Compared to the untreated cells, sHA3 treatment leads only to approximately 13% plaques reduction and sCS3 only to 6% (Figure 5B). Therefore, attachment of BCoV was not blocked.

### 3.5. Influence on Viral Penetration

**Figure 6:**
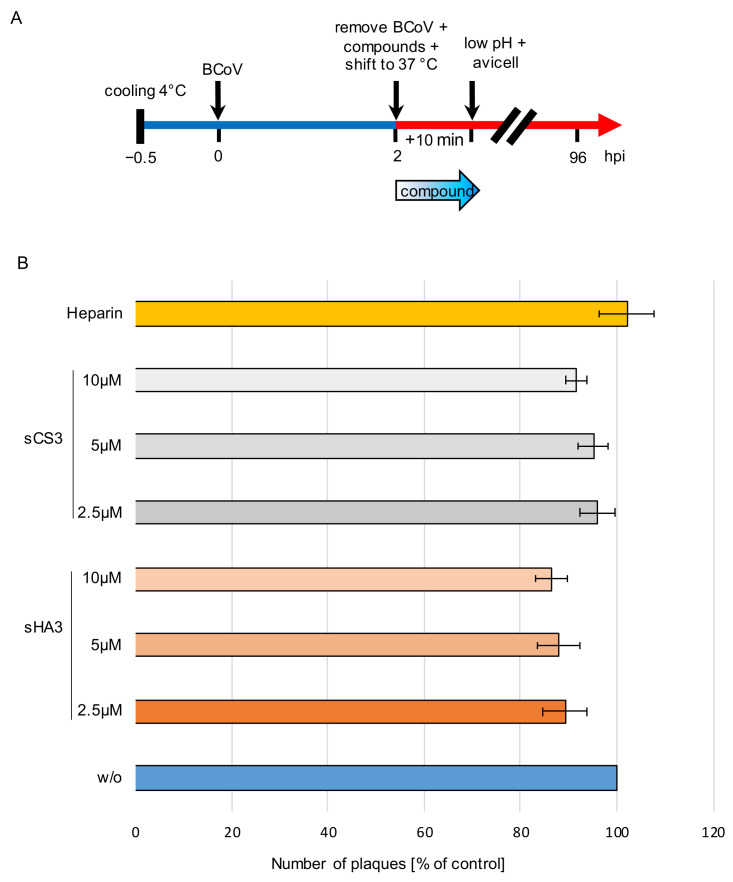
Effects of the high-sulfated GAGs on penetration. (**A**) Diagram of the experimental settings. (**B**) Prechilled PT cells were infected with prechilled BCoV (MOI 0.00005) for 2 h at 4 °C. Thereafter the cells were treated with 2.5 µM, 5 µM or 10 µM sHA3 or sCS3 or 10 µg/mL heparin or left untreated (*w*/*o*). Penetration was allowed for 10 min at 37 °C and stopped by low pH treatment (pH 3.0). Cells were overlaid with Avicel RC-581 and incubated for 4 days. Error bars on the histogram are SD from three independent experiments.

For penetration assays, prechilled PT cells were infected BCoV (MOI 0.00005) for 2 h at 4 °C. Thereafter cells were treated with 2.5 µM, 5.0 µM or 10 µM of both compounds or 10 µg/mL heparin or left untreated (Figure 6A). Penetration was allowed at 37 °C for 10 min and was stopped by low pH treatment (pH 3.0). Heparin was used as a control. Both compounds as well as heparin had only marginal effects on virus penetration. sHA3 reduced this process only up to 14 % while sCS3 reduced it up to 8% (Figure 6B). Heparin treatment did not show an effect (Figure 6B). These results imply that sHA3 and sCS3 do not prevent penetration.

### 3.6. Effect on Infectivity of Viruses

**Figure 7:**
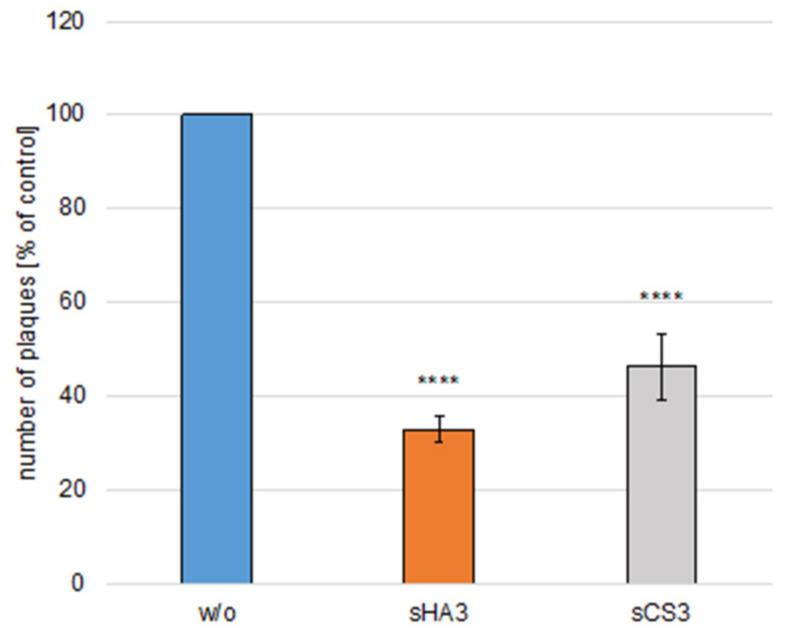
Effects of GAGs on virus inactivation. High-titer BCoV (MOI 0.005) was incubated either with sHA3 or sCS3 or with medium for 1 h at 37 °C. The mixture was diluted 1000-fold. PT cells were infected with diluted untreated virus (*w*/*o*) or pretreated virions (sHA3, sCS3) and subjected to plaque reduction assays. Error bars on the histogram are SD from three independent experiments. **** *p* < 0.001.

High titer BCoV (MOI 0.005) was incubated either with 10 µM sHA3 or sCS3 or with medium for 1 h at 37 °C. The samples were diluted 1000-fold (Figure 7). PT cells were infected with virus dilutions pre-treated with 10 µM sHA3 or sCS3 or medium (DMEM) and subjected to plaque reduction assays. As shown in Figure 7 infection, with diluted viruses with bound sHA3 or sCS3 was significantly reduced. These results demonstrated that both GAGs could inactivate BCoV by binding to the virus.

### 3.7. Effect on Viral Genome Content

**Figure 8:**
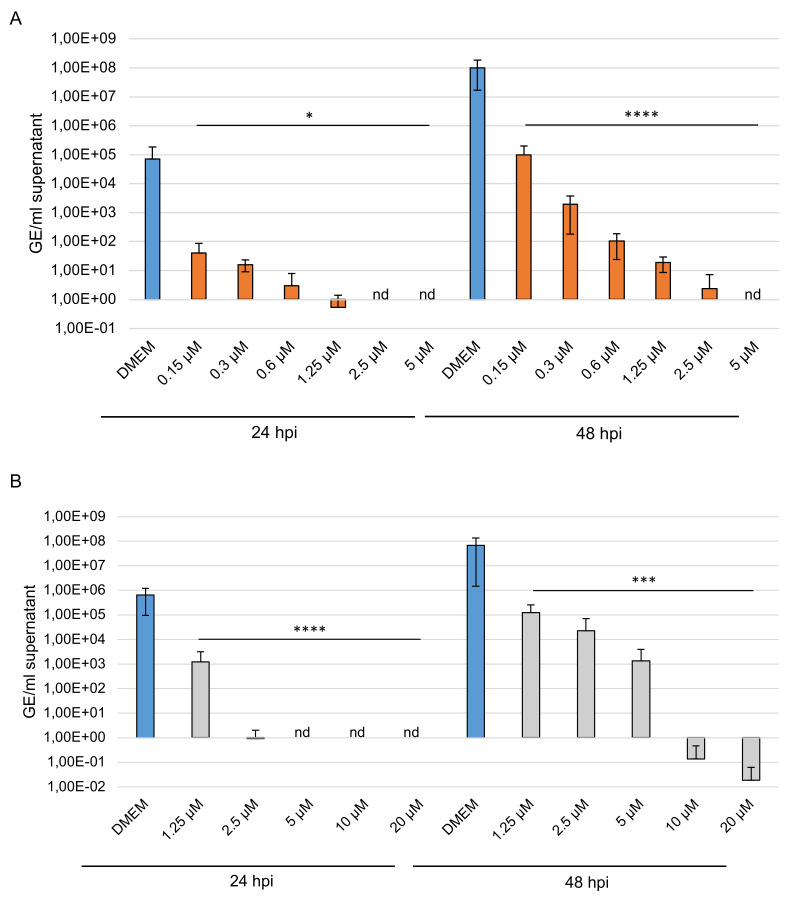
Effects of the high-sulfated GAGs on BCoV replication. PT cells were inoculated with a 2-fold dilution series of sHA3 (**A**) or sCS3 (**B**) prior to and after infection with BCoV (MOI 0.00005). At 24 and 48 hpi, the supernatants were harvested, and viral RNA was extracted. In the following, the real-time quantitative RT PCR concentration of nucleocapsid gene copy numbers was determined. Values shown represent three independent experiments. * *p* < 0.0332, *** *p* < 0.0002, **** *p* < 0.001; GE, genome equivalent.

To investigate further the effect of the two high-sulfated GAGs on virus replication, PT cells were treated with various concentrations of compounds or left untreated (DMEM) for 1 h during and after infection with BCoV (MOI 0.00001, 0.00005 and 0.0005). At 24 and 48 hpi, supernatants were harvested, and viral RNA was extracted. Viral RNA was used to quantify nucleocapsid (N) gene RNA copy numbers. The significance of the results was provided by comparison with copy number of the internal control. The results showed that even low amounts of sHA3 could block BCoV replication at 24 hpi (Figure 8A and Figure S2A,B). At 48 hpi BCoV was reduced from 1.01 × 10^8^ of the control (DMEM) to 9.83 × 10^4^ up to 3.02 and completely from 5.0 µM (Figure 8A).

The replication of BCoV was reduced at 24 hpi by sCS3 from 6.41 × 10^5^ of the control (DMEM) to 9.31 × 10^1^ (2.5 µM) compared to lesser extent at 48 hpi from 6.78 × 10^7^ (DMEM) to 1.24 × 10^5^ up to 1.33 × 10^3^ (Figure 8B). Furthermore, sCS3 prevents replication from 5.0 µM at 24 hpi, and from 10 µM at 48 hpi. These results demonstrated the high efficacy of the GAGs.

### 3.8. Efficacy on SARS-CoV-2 Replication

To determine efficiency of the compounds against SARS-CoV-2, permissive Caco-2 cells were treated with different concentrations of inhibitors before and after infection

**Figure 9:**
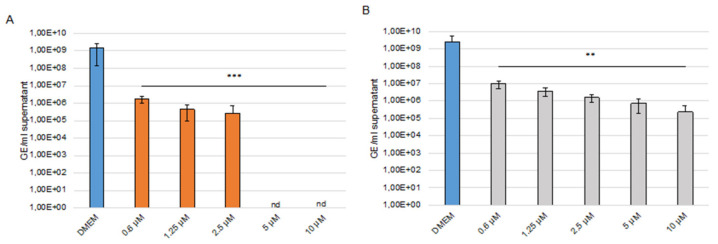
Effects of the high-sulfated GAGs on SARS-CoV-2 replication. Caco-2 cells were inoculated with a 2-fold dilution series of sHA3 (**A**) or sCS3 (**B**) prior and after infection with SARS-CoV-2 (MOI 0.001). At 24 hpi, the supernatants were harvested, and viral RNA was extracted. In the following, real-time quantitative RT PCR concentration of envelope gene copy numbers was determined. Values shown represent three independent experiments. ** *p* < 0.0021, *** *p* < 0.0002; GE, genome equivalent; nd, not detected.

With SARS-CoV-2 (MOI 0.001 and 0.0001), the CC_50_ in Caco-2 cells of sHA3 is 270 µM and of sCS3 is 265 µM, thus demonstrating that the concentrations used were not toxic (Figure S1). Supernatants were harvested at 24 hpi, viral RNA was extracted, and envelope (E) gene RNA copy numbers were determined using E gene quantitative RT PCR assay. As shown in Figure 9 (9A, sHA3; 9B, sCS3), both GAGs lead to a significant reduction in SARS-CoV-2 replication compared to the solvent control (DMEM). At 24 hpi sCS3 reduces the replication from 2.59 × 10^9^ of the control to 9.98 × 10^6^ (0.6 µM) up to 2.3 × 10^5^ (10 µM). (Figure 9B). Interestingly, the compound sHA3 was able to completely block the replication of SARS-CoV-2 at 24 (5–10 µM) hpi (Figure 9A). Comparable effects at 24 hpi were observed if a lower MOI (0.0001) was used as shown in Figure S2C.

### 3.9. Efficacy of the Compounds against SARS-CoV-2 Variants

**Figure 10:**
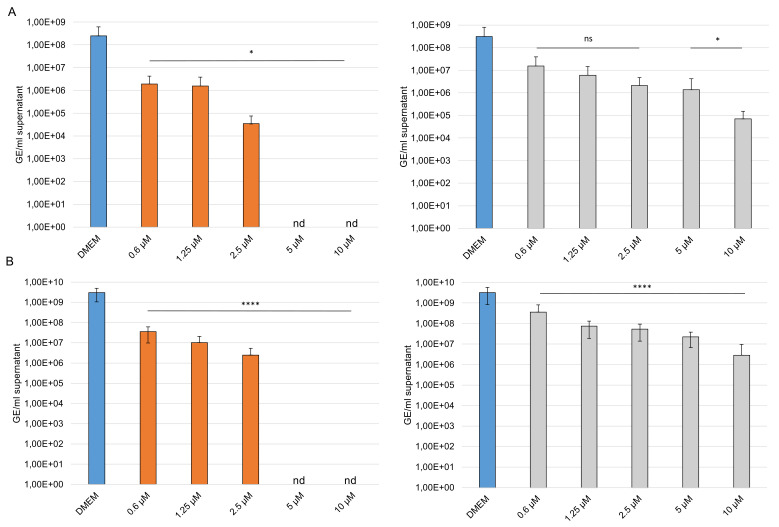
Effects of the high-sulfated GAGs on SARS-CoV-2 variants. Caco-2 cells were inoculated with a 2-fold dilution series of sHA3 (orange) or sCS3 (grey) prior and after infection with SARS-CoV-2 B.1.1.7 (**A**) or B.1.351 (**B**) (MOI 0.001). At 24 hpi the supernatants were harvested, and viral RNA was extracted. In the following, real-time quantitative RT PCR concentration of envelope gene copy numbers was determined. Values shown represent three independent experiments. * *p* < 0.0332, **** *p* < 0.001; ns, not significant; GE, genome equivalent; nd, not detected.

To gain further insights into the efficacy of our compounds, we performed analysis with SARS-CoV-2 variant B.1.1.7 (Alpha) and B.1.351 (Beta). Caco-2 cells were treated before and after infection with different concentrations of GAGs. The cells were infected with an MOI of 0.001. Supernatants were harvested at 24 hpi, viral RNA was extracted, and envelope (E) gene RNA copy numbers were determined using E gene quantitative RT PCR assay. Interestingly, both compounds inhibited the replication of the SARS-CoV-2 variants. At 20 µM sCS3 reduce the replication of the Alpha variant from 3.12 × 10^8^ of the control to 7.07 × 10^4^ (Figure 10A) and of the Beta variant from 3.27 × 10^9^ of the control to 2.87 × 10^6^ (Figure 10B). The GAG sHA3 is the most effective, because it completely prevents the replication of both variants from a concentration of 5 µM (Figure 10A,B).

## 4. Discussion

Coronaviruses (CoV) are unique RNA viruses. They have the ability to force evolution, adaption and furthermore interspecies changing [40]. It has been suggested that human CoV OC43 originated from zoonotic transmission of a bovine coronavirus (BCoV) to humans around the year 1890 [41]. The close relation between BCoV and HCoV-OC43 is also reflected by the high identity of 96% in the nucleotide gene [42]. In this study, we focus on an endemic CoV, the bovine coronavirus. This animal CoV has common characteristics with SARS-CoV-2. Both belong to the betacoronavirus genus and have a dual tissue tropism, because they infect not only the respiratory tract but also the intestine [43,44]. To date no specific drugs against CoVs are available.

Here, we describe the synthesis and antiviral activity of two glycosaminoglycans (GAGs), high-sulfated hyaluronan (sHA3) and chondroitin sulfate (sCS3). The 50% effective concentrations (EC_50_) against BCoV were 0.2996 µM for sHA3 and 1.756 µM for sCS3. These tremendous low micro molar concentrations indicate high potent antiviral activity. Furthermore, both compounds were well tolerated at concentrations lower than approximately 70 µM or 140 µM, respectively. These results were comparable with our analysis using dispiroalkane DSTP-27, as well as with tetrahalogenated benzimidazole D-ribonucleosides (BTCRB, Cl_4_RB) against human cytomegalovirus [35,45,46]. While DSTP-27 prevents entry by stable binding to cell surface, benzimidazole D-ribonucleosides are terminase inhibitors that prevent cleavage and packaging of HCMV DNA. With these compounds inhibition of viral yield increases during replication, whereas sHA3 and sCS3 showed a slight reduction only at early stage of infection. Furthermore, it was demonstrated that attachment as well as penetration were prevented neither by sHA3 nor by sCS3. These observations indicate that sHA3 and sCS3 have a completely different mode of action.

As with other Betacoronaviruses, attachment and entry of SARS-CoV-2 are mediated by the interaction of the spike glycoprotein with its cellular receptor human Angiotensin-converting enzyme-2 (ACE-2) [47]. In addition to this well-documented interaction, the spike glycoprotein has been found to bind to GAG like heparan sulfate, which is found on the surface of virtually all mammalian cells. Heparan sulfate proteoglycan (HSPG) serve as co-receptors in the process by increasing the local concentration of pathogens, so that they can more efficiently interact with their entry receptors [47,48]. Except heparan sulfate, also the GAGs heparin, heparin derivatives, and further polysaccharide sulfates have also been implicated in coronavirus entry [49]. Flow cytometry analysis showed that the adhesion of HCoV-NL63 to LLC-Mk2 cells was completely inhibited in the presence of soluble heparan sulfate [50]. These findings open the door to develop new prophylactic as well as therapeutic antiviral agents. Having in mind that the high-sulfated GAGs investigated here (sHA3, sCS3) have a chemical composition which is quite similar to the native GAGs heparan sulfate and heparin, including also the high content of sulfate groups bearing a negative partial charge. In contrast to these native GAGs, the availability of semi-synthetic high-sulfated GAGs derived from HA and CS in pharmaceutically relevant quantities is much higher due to its rather simple manufacture. In recent bioanalytical binding studies, performed by surface plasmon resonance (SPR) experiments, the high binding affinity of receptor-related proteins to different high- and medium-sulfated GAGs could be confirmed [51].

Interestingly, time of addition analysis with BCoV revealed that both compounds are active before and during infection, whereas sHA3 is even active when added after infection. In this regard, the compounds could be useful for antiviral therapy as well as prophylaxis of infection. The compound sHA3 leads to a similar decrease at all-time points. In contrast, sCS3 leads to less amounts of reduction except when it was added during infection. These results may indicate that sHA3 has the capacity to bind to cellular and viral proteins. Furthermore, sHA3 and sCS3 inactivates BCoV. This direct activity against the virus may be considered virucidal. Whether the compounds bind to the spike protein or other viral proteins has to be elucidated. The high efficacy of both GAGs was confirmed by reduction in the BCoV replication. While sHA3 leads to a complete inhibition of replication even at low concentration, sCS3 prevents replication at higher concentrations. In addition, we demonstrated that the compounds have an antiviral activity against SARS-CoV-2. Both GAGs interfere with SARS-CoV-2 replication and reduce the virus replication. Furthermore, we showed that the compounds are active against two variants, B.1.1.7 and B.1.351, thus underlining the high efficacy of both GAGs. The most efficient GAG was sHA3 that leads to a complete reduction in replication at 24 and 48 h post infection.

The different binding behavior of sHA3 and sCS3, despite approximately the same degree of sulfation, may be due to various factors. One reason may be the different molecular weight of sHA3 (83,450 g mol^−1^) and sCS3 (26,230 g mol^−1^). Another factor is the different structure of the disaccharide-repeating unit. While in the hyaluronan derivative the N-acetyl sugar is in the *gluco*, in the chondroitin sulfate it is the *galacto* conformation. As a result, a sulfate group at carbon position C-4 in sHA3 may be in an equatorial position and in sCS3 in an axial one (Figure 1). Future investigations have to confirm which structural features determine the binding affinities of both high-sulfated GAGs.

## 5. Conclusions

In summary, we demonstrated that the two high-sulfated glycosaminoglycans sHA3 and sCS3 are effective inhibitors of BCoV and SARS-CoV-2 infection. Our analysis leads to the suggestion that both compounds, sHA3 and sCS3, will be a powerful tool for development of antiviral therapy for COVID patients. Future studies are under way to (i) identify the mode of action of both compounds, (ii) determine the structural features required for antiviral activity and (iii) to identify the binding side of the compounds.

## Figures and Tables

**Table 1:** Primer and probe sequences used in real-time RT PCR.

Primer/Probe	Gene Target	Sequence (5′-3′)
BCoV	Nucleocapsid gene	
Forward		CGA TGA GGC TAT TCC GAC TAG GT
Reverse		CCT TCC TGA GCC TTC AAT ATA GTA ACC
Probe		FAM-TCC GCC TGG CAC GGT ACT CCC T-BBQ
SARS-CoV-2	Envelope gene	
Forward		ACAGGTACGTTAATAGTTAATAGCGT
Reverse		ATATTGCAGCAGTACGCACACA
Probe		FAM-ACACTAGCCATCCTTACTGCGCTTCG-BBQ

**Table 2:** Analytical data of the synthesized GAG derivatives.

Sample	sHA3	sCS3
D.S.	3.7	3.6
Mn (g mol^−1^)	56,180 (87,750)	23,180 (26,180)
Mw (g mol^−1^)	83,450 (150,040)	26,230 (40,120)
Dispersity (Đ)	1.7	1.5

sHA3: high-sulfated hyaluronan; sCS3: high-sulfated chondroitin sulfate; Mn: number-average molecular weight, Mw: weight-average molecular weight, determined by gel permeation chromatography (GPC) values as determined by laser light scattering detection and refraction index (RI) detection (in parentheses); Dispersity (Đ = M_w_/M_n_) detected by GPC (values calculated from RI detection).

**Table 3:** Antiviral activities of high sulfated glycosaminoglycans against BCoV.

Compound	Mean EC_50_ (µM) ^a^	Mean CC_50_ (µM) ^b^	SI (µM)
	Plaque Reduction	Cell Proliferation	CC_50_/EC_50_
sHA3	0.2996 ± 2.27	69.710 ± 0.80	232.68
sCS3	1.7156 ± 1.08	141.360 ± 0.04	82.39

^a^ The EC_50_ was defined as the concentration of compound that resulted in a 50% plaque reduction compared to the untreated control. ^b^ The CC_50_ was defined as the concentration of compound that resulted in a 50% reduction in the cell proliferation. Values represent means ± standard deviation from three independent experiments.

## Data Availability

The data presented in this study are available on request from the authors. The data are not publicity available due to restricted access to the server of the Charité-Universitätsmedizin Berlin and INNOVENT e.V.

## Supplementary Materials

The following are available online at https://www.mdpi.com/article/10.3390/v14020413/s1, Figure S1: Cytotoxicity of compounds in Caco-2 cells. Figure S2: Efficacy of different virus concentrations.
