# Supplementary material for: High-Sulfated Glycosaminoglycans Prevent Coronavirus Replication
Source: Viruses. 2022 Feb 17;14(2):413. doi: 10.3390/v14020413 (PMC8877876; doi:10.3390/v14020413)
Supplement: Supplementary file 1 [file viruses-14-00413-s001.zip › viruses-1600272-supplementary.pdf]

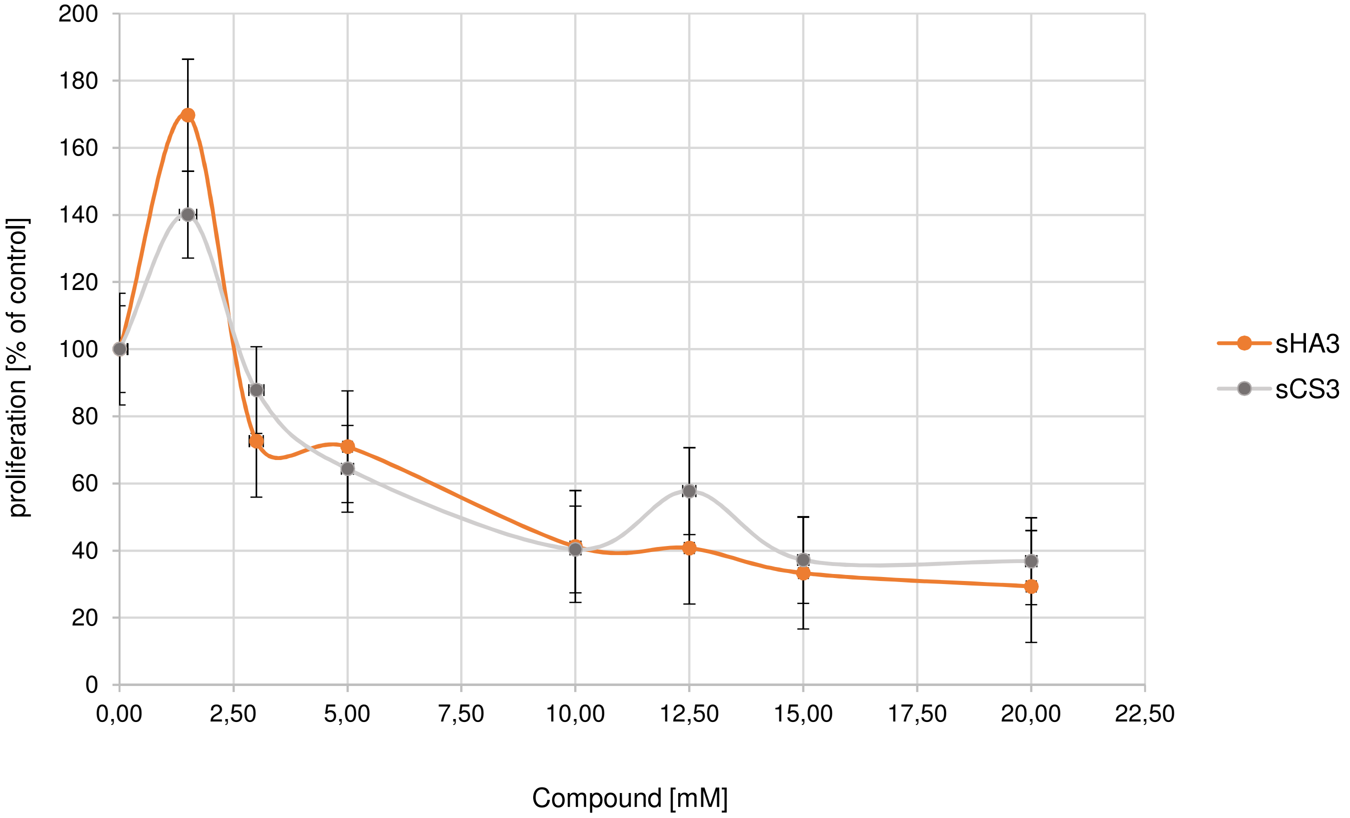


**Figure S1**: Cytotoxicity of the compounds in Caco-2 cells. Various concentrations of sHA3 or sCS3 (mM) were added to Caco-2 cells. At 24 h, XTT cell proliferation assay was performed. Values are represented as percentage of untreated control cells. Data are mean values from three independent experiments. Error bars represent the standard deviation.

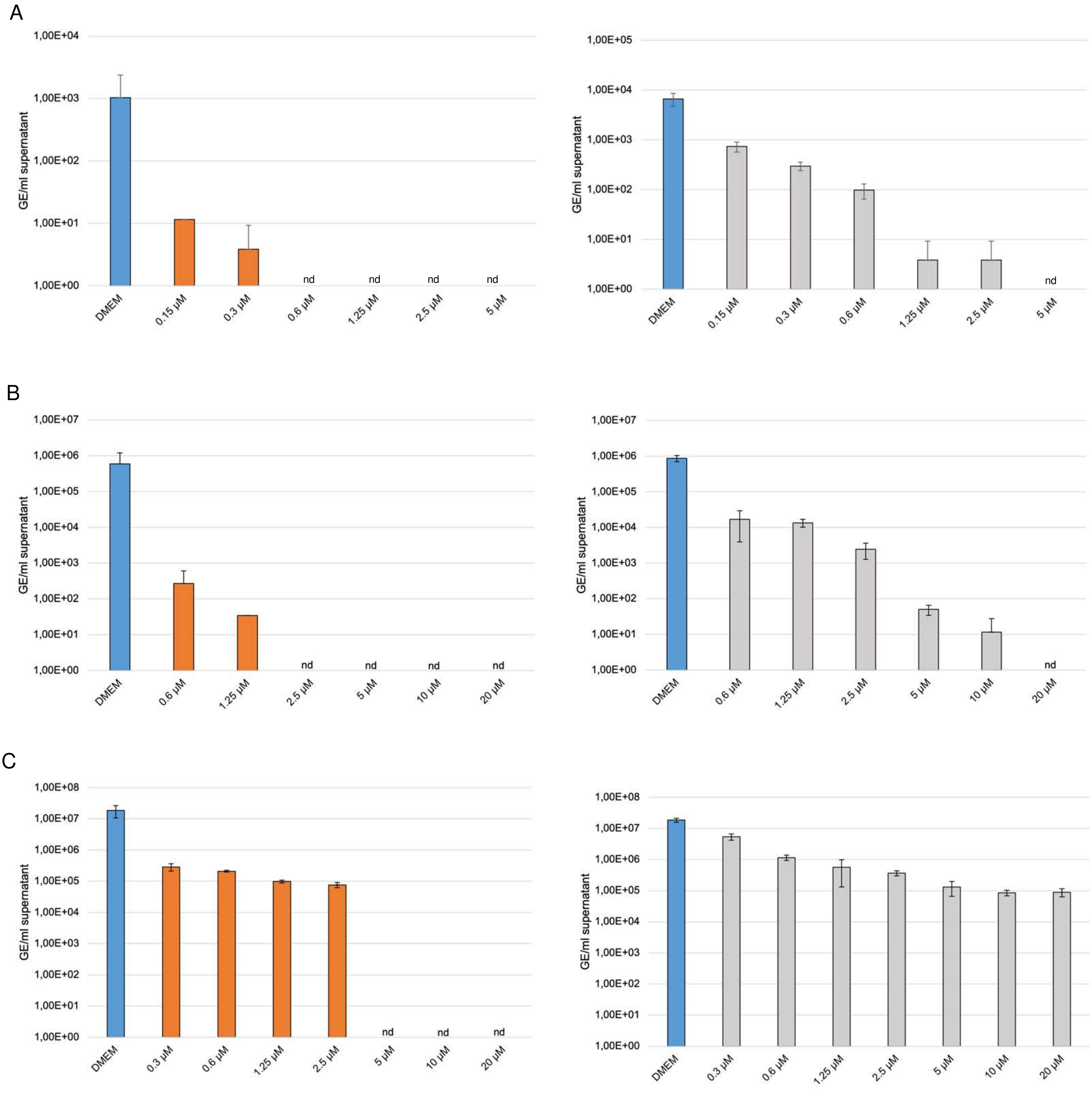


**Figure S2**: Efficacy of different virus concentration. PT cells (A,B) or Caco-2 cells (C) were inoculated with a 2-fold dilution series of sHA3 (orange) or sCS3 (grey) prior and after infection with BCoV MOI 0.00001 (A) or 0.0005 (B) and SARS-CoV-2 MOI 0.0001 (C). At 24 hpi the supernatants were harvested and viral RNA was extracted. In the following, real-time quantitative RT PCR concentration of nucleocapsid or envelope gene copy numbers was determined. Values shown represent three replicas. GE, genome equivalent; nd, not detected
